# Population shifts in some faeces and rumen bacteria profiles and subsequent blood LPS and lactate concentrations in lambs in the early period of subacute ruminal acidosis

**DOI:** 10.1002/vms3.978

**Published:** 2022-10-26

**Authors:** Ali Abbas Nikvand, Mohammad Nouri, Darioush Gharibi, Rahman Rakhshandeh

**Affiliations:** ^1^ Department of Clinical Sciences, Faculty of Veterinary Medicine Shahid Chamran University of Ahvaz Ahvaz Iran; ^2^ Department of Pathobiology, Faculty of Veterinary Medicine Shahid Chamran University of Ahvaz Ahvaz Iran

**Keywords:** lactate, lactic acid bacteria, LPS, rumen bacteriology, SARA

## Abstract

**Background:**

It is known that ruminal acidosis can induce harmal population shifts in some ruminal bacteria profiles. However, there is little information related to alterations in faecal and ruminal bacterial communities and relevant serum lipopolysaccharide (LPS) in sheep with subacute ruminal acidosis (SARA).

**Objectives:**

This study aimed to investigate alterations in the defined faecal and ruminal bacteria profiles and serum LPS and blood lactate concentrations in lambs with empirically induced SARA.

**Methods:**

Fifteen lambs were served and undergone to induce SARA during a 7‐day period. Faecal and ruminal samples were taken to measure the pH and to perform the bacteriological works at 0 (just before induction), 8, 9, and 10 days of the challenge. Blood samples were collected to determine the serum LPS and lactate levels. The rumen and faecal samples were cultured to specify colony‐forming units (CFU) for *Escherichia coli*, *Streptococcus* Group D (SGD), and lactic acid bacteria (LAB).

**Results:**

Serum LPS value had no significant increase in the affected lambs with SARA. Significant increasing trends were observed in faecal *E. coli* and LAB populations (*p* < 0.01). Rumen bacteriology revealed a rising trend for LAB and a falling trend for SGD populations (*p* < 0.01).

**Conclusion:**

: Unlike cattle, LPS appears to be of minor importance in the pathogenesis of SARA in sheep. The increased ruminal and faecal LAB (4.00 × 10^7^ CFU/ml or g) are proposed as valuable biomarkers for improving nutritional strategy and screening SARA in lambs.

## Graphical abstract

Fifteen lambs were undergone to induce subacute ruminal acidosis (SARA) and the rumen and fecal samples were cultured to specify CFU for *E. coli*, *Streptococcus* Group D, and lactic acid bacteria (LAB). Significant increasing trends were observed in the fecal and ruminal LAB populations. The increased ruminal and fecal LAB are proposed as valuable biomarkers for improving nutritional strategy and screening SARA in lambs.

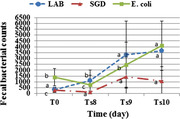

## 1 INTRODUCTION

Ruminal acidosis is a prevalent nutritional disorder in ruminants. This disorder increases herd mortality, dramatically reduces weight gain, and complicates nutritional strategies in cattle and sheep. Subacute ruminal acidosis (SARA), also known as subclinical acidosis, is economically more important than its acute form, and it is often able to affect a significant proportion of herd (Constable et al., 2017). SARA has been defined as a sequel of feeding ruminants (formerly on predominantly forage‐based rations) with high‐grain rations (Smith, 2015).

Several studies have focused on the epidemiology and pathophysiology of the disease in cattle, sheep, and goat (Bramley et al., 2008; Lee et al., 2019; F. Li et al., 2017; Plaizier et al., 2017a). SARA is generally defined as a depressed rumen pH value for extended periods (Kleen & Cannizzo, 2012; Plaizier et al., 2008). It has been considered by several researchers as a rumen with pH below 5.80 (5.80–5.20) (Morgante et al., 2009; Sharma et al., 2009). Rumen pH has been reported to vary in SARA, with the lowest value recorded 2–4 h following concentrate consumption (Harrison et al., 1989). As a part of SARA pathogenesis, the escape of large amounts of fermentable carbohydrates from the rumen and small intestine could lead to extensive fermentation in the hindgut during SARA. Hindgut fermentation was also shown to result in reduced digesta and faecal pH in SARA (Gressly et al., 2011; S. Li et al., 2012). Furthermore, S. Li et al. (2012) claimed that hindgut fermentation might alter the faecal bacterial communities during SARA.

Several experiments have been conducted on rumen (Khafipour, Krause, et al., 2009; Plaizier et al., 2017a) and faecal (Mao et al., 2012) bacteria communities in dairy cows during grain‐induced SARA; one of these studies reported a dominant rumen flora of *Streptococcus bovis* and *Escherichia coli* (Khafipour, Krause, et al., 2009). Despite the rumen and faecal bacteriological studies during SARA in cow, little information is available on changes in rumen and faecal bacterial density and diversity in sheep or lambs with SARA (F. Li et al., 2017).

The *E. coli* is a predominant rumen bacterial flora at the normal pH value. Following consumption of fermentable carbohydrates and reduction of the rumen pH, due to overgrowing in *Streptococcus* spp. proliferation and excessive volatile fatty acids (VFAs) accumulation in the rumen, it is expected to kill the Gram‐negative rumen flora such as *E. coli* and subsequently blood lipopolysaccharide (LPS) increment (Constable et al., 2017; Khafipour, Krause, et al., 2009). On the other hand, it has been shown that the numerous species of lactate‐producing and lactate‐using bacteria can adapt to a decreased rumen pH (Smith, 2015).

Although the ruminal pH assessment is a valuable method for diagnosing SARA, it has been shown that the changes in the communities and the abundances of rumen bacteria are more stable than the ruminal pH changes over 2–4 h after concentrate feeding in lambs (F. Li et al., 2017). It has also been reported that abundances of several rumen cellulolytic bacteria could be used as a potential biomarker for SARA screening in lamb flock (F. Li et al., 2017). Therefore, a better understanding of the rumen and faecal bacterial structures during SARA in sheep can be conducive to diagnosing or specifying the risk of SARA and to developing a better nutritional strategy.

The major pathogenesis of SARA is related to VFAs accumulation, pH depression, bacterial population changes, and subsequently LPS releasing into the bloodstream (Constable et al., 2017; Plaizier et al., 2008). It has been revealed that SARA induction via feeding high‐grain rations is associated with elevated levels of free bacterial LPS in the ruminal and intestinal digesta in dairy cows (Plaizier et al., 2012). The LPS or endotoxin could translocate into blood and result in many complications, including inflammatory responses, ruminal stasis, poor blood supply in tissue, and weakness (Pulina & Bencini, 2004). However, blood LPS changes and its association with the rumen and faecal bacterial perturbations during SARA in sheep are still unclear. Therefore, the current study aimed to (1) investigate the change in the density of certain ruminal and faecal bacterial communities and (2) evaluate their relationship with the blood LPS and lactate concentrations in experimentally induced SARA in lambs.

## 2 MATERIALS AND METHODS

This interventional, clinical‐laboratory field trial was performed on 15 six‐month‐old Arabian lambs (12 males and three females) with an average live weight of 32.50 ± 3.50 kg in the Veterinary Teaching Hospital of Shahid Chamran University of Ahvaz (Khuzestan province, southwest Iran) in September 2018.

### 2.1 Study periods

The study course comprised a 1‐week on basal diet and 1 week of induction (adaptation) period which were followed by a 3‐day period of SARA status.

### 2.2 Period on basal diet

After determining the dry matter (DM) content of dietary ingredients (crushed corn 89.40%, alfalfa 86.20%, and wheat straw 92.20%), in the first week of the study, the lambs were individually fed with a basal diet at roughly 3.00% body weight with a 1:4 ratio of corn to forage (220 g crushed corn, 430 g straw, and 400 g alfalfa) on a DM basis. Water access was free. The diurnal rations were offered in three servings at 8:00, 14:00, and 19:00 h, such that the corn was fed in the morning (8:00) and in the afternoon (14:00) (Nikvand et al., 2020).

### 2.3 Induction (adaptation) period and sampling

Just before the induction (zero day), as the pre‐treatment sampling, the blood, rumen fluid, and faecal samples were taken from the lambs. Afterwards, the crushed corn was gradually added to the basal diet on a daily basis at 5.00% weight of DM ration (42.00 ± 8.00 g), and the same amount of straw was simultaneously deducted. Each lamb was individually fed with crushed corn (Nikvand et al., 2020). In all animals, on the 7th day of SARA induction, when the amount of crushed corn increased to 52.80% (566 ± 92.00 g) of the ration, ruminal pH was reduced to a range of 5.60–5.70 between 4 and 7 h after morning feeding (for at least 3 h per day); this pH range represented SARA (Constable et al., 2017; Morgante et al., 2009; Penner et al., 2009). A rumen pH threshold below 5.80 has been widely applied to determine SARA severity in sheep (Penner et al., 2009).

*Rumen sampling*: After rumen content sampling with an orogastric tube (Lee et al., 2019; F. Li et al., 2017), pH was immediately specified by a pen type digital pH meter (AZ8686; AZ Instrument Corp., Taichung, Taiwan). The ruminal pH was measured twice a day between 4 and 7 h after morning feeding during induction and SARA periods. To avoid oral bacteria and saliva contamination of the rumen samples, a disinfected large‐bore orogastric tube (1.50 cm of the inside diameter and 40.00 cm in length) was primarily inserted in the upper oesophagus. Next, a disinfected small‐bore stomach tube (1.00 cm of the inside diameter) was passed through the larger tube into the rumen. The first amounts (approximately 50.00 ml) of the gushed out fluid samples were further discarded due to the possible oral bacteria and saliva contamination. It is notable that prior to use, the orogastric tubes were washed with Betadine iodine 10.00% and then rinsed with sterile saline solution.

*Faecal sampling*: A faecal specimen was directly obtained from the rectum using disposable gloves. In this way, 10 g of the specimen was transferred into a sterile falcon tube and referred to the laboratory for bacteriological testing; also, 5 g was mixed with the same amount of distilled water, and its pH was immediately determined by the digital pH meter (Johnson & Rossow, 2018).

*Blood sampling*: Two blood samples were also taken from the jugular vein. To measure the blood pH and certain biochemical variables, a heparinized blood sample was taken by a 2 ml‐sterile syringe manually soaked with 0.05 ml of sodium heparin (125 IU/ml blood; Caspian Co., Rasht, Iran). Venous blood gas analysis has been applied to evaluate some blood variables regarding ruminal acidosis in cows (Marchesini et al., 2013). For serum separation, after clotting, anticoagulant‐free blood samples were centrifuged at 3000 rpm for 10 min; then, the harvested sera were stored in 1.50 ml sterile micro‐tubes and frozen at – 70˚C until further use.

### 2.4 SARA period

Over the 3 consecutive days of SARA (which were days 8, 9, and 10 of the challenge), each lamb was fed with 566 ± 92 g of crushed corn, 98 ± 16 g of wheat straw, and 400 g of alfalfa. To assess the rumen bacteriology, 10 ml of the rumen fluid sample was daily taken from each animal, transferred to a sterile falcon tube, and referred to bacteriological evaluations in less than 1 h. The pH was determined with the rest of the ruminal sample. The blood samples were further obtained to analyze the blood biochemical parameters. Every 3 days of the SARA period, a faecal specimen was taken from each lamb for pH determination and bacteriology.

### 2.5 Bacteriology of rumen samples

The population changes of the rumen and faecal *E. coli*, *Streptococcus* Group D (SGD), and lactic acid bacteria (LAB) were studied. After homogenization, the rumen fluid samples were initially diluted at 1:10,000 (10^−4^) level using a sterile phosphate buffer solution (PBS; Merck, Darmstadt, Germany). After that, 50 µl of this dilution was inoculated and cultured in Eosin Methylne Blue (EMB) (Biolab, Budapest, Hungary), Bile Esculin Azide agar (BEA; Merck), and De Man, Rogosa and Sharpe agar (MRS; Scharlau, Sentmenat, Spain) media. EMB, BEA, and MRS culture media were employed to count *E. coli*, SGD (including *Enterococcus* spp., *S. bovis*), and LAB populations, respectively (Markey et al., 2013). The rumen fluid samples at a diluted level of 1:100,000 (10^−5^) were further cultured in Tryptic Soy Agar (TSA; Merck), a medium for anaerobic bacterial count. Plates of TSA and MRS media were incubated under anaerobic conditions at 37˚C for 24–48 h. Ultimately, the bacterial colonies were enumerated, and colony‐forming units (CFUs) per ml of the rumen fluid samples were calculated as follows:

CFU/ml of the rumen fluid sample = Number of colonies × dilution factor (the inverse of the dilution) × 20 (Markey et al., 2013).

### 2.6 Bacteriology of faecal samples

At first, 1 g of a faecal sample was mixed with 9 ml of sterile PBS solution to obtain a homogeneous and diluted solution at 1:10 (10^−1^) level. Subsequently, a diluted sample was prepared at 1:10,000 (10^−4^) level. Then, 50 µl of this dilution was inoculated and cultured in each of EMB, BEA, and MRS media. The faecal samples (50 µl), diluted at 1:100,000 (10^−5^) level, were further cultured in TSA medium for total anaerobic bacterial count. The culture conditions and CFU/g calculation for faecal samples in different media for rumen fluid samples were the same as mentioned above (Markey et al., 2013).

### 2.7 Blood biochemical analyses

Blood biochemical variables such as lactate, bicarbonate, anion gap, and blood pH were immediately measured after sampling by use of a blood gas analyzer and its cartridge pack (EDAN; Edan Instruments Inc., Shenzhen, China) (Johnson & Rossow, 2018). A commercial bacterial LPS ELISA kit (Shanghai Crystal Day, Biotech, China) was utilized to measure serum LPS concentration according to the kit instruction using an Accu Reader Microplate reader (Metertech, Taipei, Taiwan) (Johnson & Rossow, 2018).

### 2.8 Data analysis

Shapiro–Wilk test showed that some but not all data had a normal distribution. For the normally distributed data, a repeated measure analysis of variance (ANOVA) was employed to evaluate the overall trend of data changes over the four time points of sampling; these time points consisted of days 0 (just before induction), 8, 9, and 10 of challenge in the studied lambs. For abnormally distributed data (e.g., SGD bacterial data), a non‐parametric Friedman test was also employed. Using Pearson test, the correlations of the serum LPS and blood lactate changes with the rumen and faecal bacterial population were also calculated. The level of significance was set to *p* < 0.05. The analyses were performed using SPSS statistic software (version 24.0; IBM Corp., Armonk, USA).

## 3 RESULTS

### 3.1 Ruminal and faecal pH variations

According to the results, on the seventh day of induction of SARA, the rumen pH of all lambs decreased to a range of 5.60–5.70, which was considered SARA. Statistical analysis showed that the changes in the faecal pH of the lambs were associated with a significant falling trend during days 0 (just before induction), 8, 9, and 10 of the challenge (*p* < 0.01), (Table 1); therefore, on the second and third days of SARA, the mean faecal pH was reduced to 6.29 ± 0.20. A strong positive correlation was observed between the mean rumen pH changes and the mean faecal pH changes during the times of induction and SARA phases in the studied lambs (*r* = 0.974, *p* < 0.001).

**TABLE 1:** Diurnal variations of the ruminal and faecal pH (mean ± SD) during induction and subacute ruminal acidosis (SARA) phases in the studied lambs

pH	Induction phase (day)							SARA phase (day)		
	0	2	3	4	5	6	7	8	9	10
Rumen	7.10 ± 0.20^a^	6.62 ± 0.20^a^	6.30 ± 0.10^ab^	6.20 ± 0.10^ab^	5.90 ± 0.20^ab^	5.90 ± 0.10^ab^	5.80 ± 0.10^ab^	5.64 ± 0.10^ac^	5.62 ± 0.10^abc^	5.70 ± 0.10^ac^
Faecal	7.30 ± 0.20^a^	7.10 ± 0.20^a^	6.80 ± 0.20^a^	6.80 ± 0.20^a^	6.60 ± 0.20^ab^	6.60 ± 0.10^ab^	6.50 ± 0.10^ab^	6.40 ± 0.30^ac^	6.29 ± 0.20^abc^	6.29 ± 0.20^abc^

*Note*: Different superscript letters on each time point in the rows denote significant difference in pH values among the times (*p* < 0.01).

### 3.2 Blood parameter variations

Repeated measure ANOVA test, showed that induction of SARA did not significantly change the serum LPS levels at either time points (days 0, 8, 9, and 10 of the challenge) in the studied lambs (Table 2). Based on repeated measure ANOVA, no significant changes were detected in the studied lambs regarding blood lactate, bicarbonate, anion gap, and pH values at either time points during the early period of SARA (Table 2).

**TABLE 2:** Mean ± SD serum lipopolysaccharide (LPS) and blood lactate, bicarbonate, anion gap, and pH values of studied lambs during subacute ruminal acidosis (SARA)

Blood parameters	Before induction	Days of SARA		
	0	8	9	10
LPS (pg/ml)	3.40 ± 2.50	3.45 ± 3.36	5.10 ± 4.10	4.40 ± 3.30
Lactate (mg/dl)	11.80 ± 9.50	10.00 ± 9.70	13 ± 11.5	8.10 ± 6.90
Bicarbonate (meq/L)	25.00 ± 2.40	25.00 ± 2.90	24.70 ± 2.10	25.50 ± 2.50
Anion gap (mmol/L)	8.30 ± 3.00	8.50 ± 3.00	9.80 ± 8.10	8.10 ± 3.60
pH	7.43 ± 0.04	7.43 ± 0.05	7.44 ± 0.03	7.44 ± 0.05

*Note*: No significant difference was found among the sampling time points in the blood parameters (*p* > 0.05).

### 3.3 Rumen bacteriology results

**FIGURE 1:**
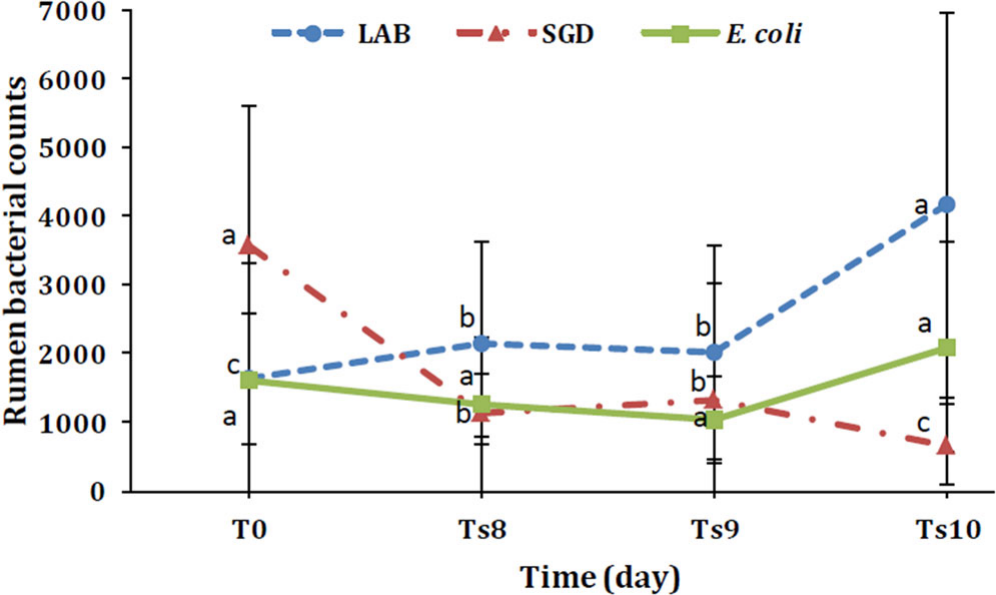
Changes in the rumen bacterial counts (CFU/ml) × 10^4^ (means) in the studied lambs with induced‐SARA. LAB, Lactic acid bacteria; SGD, *Streptococcus* group D; T0, before induction; Ts8–Ts10, SARA period Different superscript letters on each time point denote significant differences among times (*p* < 0.01).

The data analysis showed a significant falling trend in ruminal anaerobic bacterial count over the early period of SARA (*p* < 0.01). Rumen bacteriology revealed a rising trend for LAB and a falling one for SGD population (*p* < 0.01), (Table 3). Rumen *E. coli* population slightly increased during this short period of SARA (*p* > 0.05) (Figure 1).

**TABLE 3:** The means ± SD levels of changes in the rumen bacterial counts (colony‐forming units [CFU]/ml) of lambs during subacute ruminal acidosis (SARA)

Bacteria	Before induction	Days of SARA		
	0	8	9	10
AnTBC (10^5^)	20213 ± 9512^a^	27242 ± 18,424^a^	1999 ± 1404^b^	7780 ± 5750^b^
SGD (10^4^)	3554 ± 2037^a^	1112 ± 1132^b^	1221 ± 1708^b^	658 ± 581^c^
LAB (10^4^)	1637 ± 939^c^	2150 ± 1482^b^	2005 ± 1558^b^	4153 ± 2793^a^
*E. coli* (10^4^)	1696 ± 1610^a^	1264 ± 462^a^	1046 ± 633^a^	2090 ± 1526^a^

*Note*: Different superscript letters in each row denote significant differences among times (*p* < 0.01).

Abbreviations: AnTBC, anaerobic total bacterial count; LAB, lactic acid bacteria; SGD, *Streptococcus* group D.

### 3.4 Faecal bacteriology results

**FIGURE 2:**
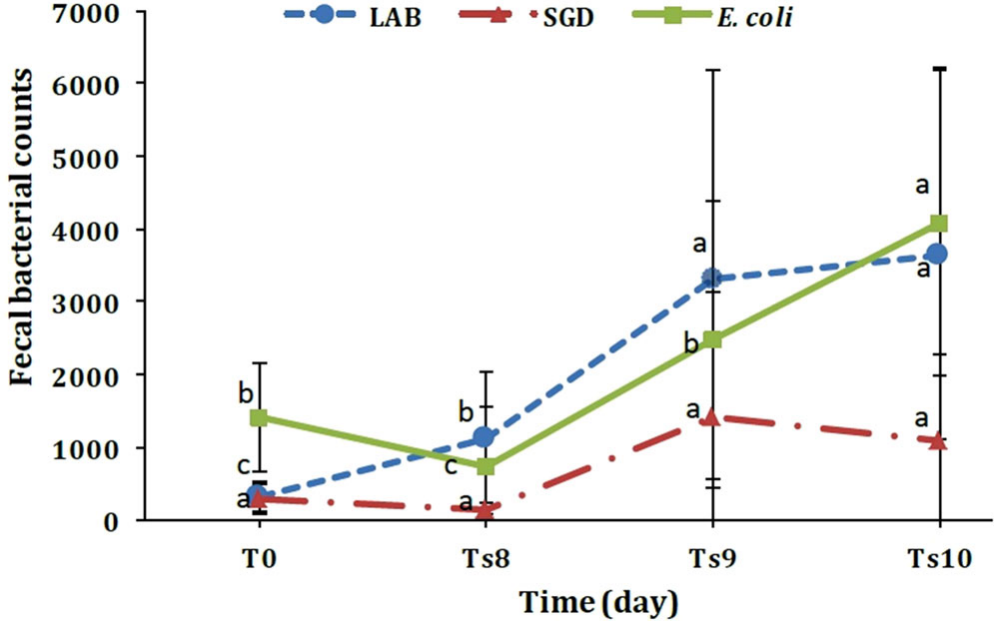
Changes in the faecal bacterial counts (colony‐forming units [CFU]/g) × 10^4^ (means) in the studied lambs with induced‐SARA. LAB, Lactic acid bacteria; SGD, *Streptococcus* group D; T0, before induction; Ts8–Ts10, SARA period Different superscript letters on each time point denote significant differences among times (*p* < 0.01).

Significant falling trends were found in faecal anaerobic bacterial densities during early period of SARA (*p* < 0.01). Non‐parametric Friedman test showed that the faecal SGD populations non‐significantly increased on days 8, 9, and 10 of SARA in comparison with day 0 (*p* > 0.05) (Figure 2). A significant rising trend was further seen in LAB counts during SARA (*p* < 0.01). The faecal *E. coli* count was associated with a significant increase on days 9 and 10 of SARA compared to day 0 (*p* < 0.01) (Table 4).

**TABLE 4:** The means ± SD levels of changes in the faecal bacterial counts (colony‐forming units [CFU]/g) of lambs during subacute ruminal acidosis (SARA)

Bacteria	Before induction	Days of SARA		
	0	8	9	10
AnTBC (10^5^)	23,613 ± 8849^a^	6026 ± 4403^b^	4244 ± 2129^b^	6346 ± 3704^b^
SGD (10^4^)	296 ± 206^a^	145 ± 68.00^a^	1421 ± 1708^a^	1086 ± 1199^a^
LAB (10^4^)	321 ± 213^c^	1129 ± 901^b^	3320 ± 2882^a^	3645 ± 2536^a^
*E. coli* (10^4^)	1397 ± 742^b^	733 ± 812^c^	2475 ± 1918^b^	4092 ± 2116^a^

*Note*: Different superscript letters in each row denote significant differences among times (*p* < 0.01).

Abbreviations: AnTBC, anaerobic total bacterial count; LAB, lactic acid bacteria; SGD, *Streptococcus* group D.

Pearson correlation test showed no significant association between serum LPS levels with the rumen and faecal *E. coli* counts and other rumen bacterial populations. Additionally, no significant correlation was observed between blood lactate level and rumen bacterial changes over the course of the experiment.

## 4 DISCUSSION

There is no research regarding the changes in rumen bacterial community in the context of rumen pH changes and its association with blood LPS in sheep on a SARA induction diet. It has been shown that SARA in cattle is correlated with change in the ruminal and faecal bacterial community and production of LPS (Plaizier et al., 2017a). It has further been reported that rumen‐derived bacterial LPS plays a prominent role in the pathogenesis of SARA in cattle (Guo et al., 2017; Plaizier et al., 2012).

Following the induction period, a reduction occurred in the rumen pH to the range of 5.60–5.70, which is indicative of SARA in the studied lambs. In this regard, a rumen pH threshold value of 5.8 was considered to define SARA in sheep (Penner et al., 2009), goats (Dong et al., 2013), and dairy cows (Mao et al., 2012). The results showed that the decrease in the rumen pH of lambs to 5.70–5.60 reduced the faecal pH from 7.30 ± 0.20 to 6.29 ± 0.20 on the second and third days of SARA. Supporting our results, Mao et al. (2012) reported a reduction in faecal pH from 7.15 to 6.40 during SARA in dairy cows. This may be attributed to hindgut fermentation of an escaped massive unstructured carbohydrate from the rumen (Gressley et al., 2011)

Induction of a short period of SARA did not significantly change the serum LPS value over the trial in the studied lambs. In agreement with our study, a previous study reported that feeding with two diets containing 50% and 25% concentrate did not significantly change the serum LPS values in goats (Huo et al., 2014). It has been also claimed that there is limited evidence of increased circulatory LPS during acidosis, which might be ascribed to measurement errors or rapid blood clearance (Plaizier et al., 2012). In conflict with our results, an increased plasma LPS value was further reported following grain‐induced SARA in Holstein dairy cows (Guo et al., 2017; Khafipour, Li, et al., 2009). The rumen‐derived endotoxins are mainly metabolized by hepatic macrophages (Andersen, 2003; Emmanuel et al., 2007). In dairy cows, the bovine liver function decreased following hepatic lipidosis, which is quite frequent (Andersen, 2003); in the authors’ opinion, this may be a possible reason for the difference between sheep and cows regarding the ability of the liver to remove blood LPS. Interestingly, however, SARA did not increase serum LPS values in the studied lambs; it may also be attributed to the short duration of SARA in the studied lambs. Therefore, this necessitates more research for a more definitive conclusion.

The SARA induction did not lead to significant changes in lactate, bicarbonate, anion gap, and blood pH values ​​in the studied lambs. Similar to our study, previous investigations reported no significant changes in blood lactate and pH values​​ in dairy cows with SARA (Daschner et al., 2015; S. Li et al., 2012). It was previously observed that blood lactate, bicarbonate, and pH levels were within normal ranges during SARA in cattle (Brown et al., 2000), which is in agreement with our results. Pathology of SARA is attributable to the accumulated ruminal VFAs. In the pH range of 5.80–5.50, lactate‐using bacteria are dominant over lactate‐producing ones. Lactate‐using bacteria are able to convert rumen lactate to pyruvate as a substrate for gluconeogenesis (Hernandez et al., 2014). Therefore, blood lactate level is not expected to increase during SARA. In the present study, the significant reduction in the rumen *Streptococcus* spp. population as a lactate producer bacterium supports the above explanations. During SARA, both overproduction and reduced absorption of VFAs occur in the rumen. The rumen VFAs, which often contain acetic, propionic, butyric, and a small number of lactic acids, are metabolized to some beneficial energy metabolites in the rumen wall and liver after being absorbed; accordingly, they cannot affect the blood acid‐base‐related components, including pH, bicarbonate, and anion gap (Constable et al., 2017).

Our study showed that the density of ruminal anaerobic bacteria significantly decreased within SARA in lambs, which is consistent with the results of Plaizier et al. (2017b). They observed reductions in the richness and diversity of ruminal bacteria in SARA challenge in dairy cows. Rumen bacteriology revealed an increase in LAB, a decrease in SGD, and no significant changes in *E. coli* populations in the studied lambs. Some of the foregoing findings were supported by Goad et al. (1998), who reported elevated ruminal *Lactobacilli* counts in steers on a SARA induction diet. A highly abundant *Lactobacillus* population was further observed in cows fed on SARA‐inducing diets (Wang et al., 2014). In accordance with the present results, a reduction in rumen *Streptococcal* population was reported in dairy cows on a SARA‐inducing diet (Mao et al., 2013). Despite the significant increase in the rumen LAB population, either producers or utilizers of lactate, no notable changes were found in the blood lactate level of the studied lambs. This is possibly explained by the fact that major lactate‐utilizing bacteria such as *Megasphaera elsdenii* and *Selenomonas Ruminantium* are still not suppressed in the rumen pH of 5.70–5.60. (Constable et al., 2017; McCann et al., 2016; Meissner et al., 2010).

The abundance of ruminal fluid *E. coli* isolated from the current studied lambs on a SARA‐inducing diet did not significantly change from before the induction (1.60 × 10^7^) to the third day of SARA (2 × 10^7^ CFU/ml). It may be related to the short duration (3 days) of SARA. Similar findings were reported by Khafipour et al. (2011) in dairy cows with SARA. Serum LPS levels might result from the lysis of rumen Gram‐negative bacteria other than *E. coli* (Andersen et al., 1994); however, the unaffected serum LPS values in the lambs might be ascribed to the slight changes in the rumen *E. coli* population during SARA.

The faecal bacteriology also revealed a significant rising trend in *E. coli* population during SARA days. In line with our findings, an increased faecal *E. coli* count was recorded in cows on a SARA‐inducing diet (McCann et al., 2016; Plaizier et al., 2017b). The number of faecal *E. coli* significantly increased in the studied lambs on the third day of SARA (4 ± 2 × 10^7^ CFU/g) compared to day 0 (1.40 ± 0.70 × 10^7^). In agreement with this finding, Diez‐Gonzalez et al. (1998) reported an increase in *E. coli* count from 2 × 10^4^ to 6.30 × 10^6^ CFU/g of colon contents in cows with SARA. Similarly, a 1000‐fold decline was also detected in *E. coli* population in hindgut when the cattle were switched from a high‐corn diet to a forage diet within 5 days (Callaway et al., 2009). Based on the results, it seems that during SARA, *E. coli* proliferation occurs more often in the colon than in rumen. This finding is further supported by Emmanuel et al. (2007) who found that LPS was mainly absorbed from the colons in cows on a SARA‐inducing diet.

The SARA induction resulted in a significant rising trend (from 0.30 × 10^7^ to 3.60 × 10^7^ CFU/g) in the number of faecal LAB community in the studied lambs. This suggests that the bacterial community might have a prominent metabolic potential to transfer from forage to high‐concentrate diets with the occurrence of SARA. Consistent with our results, it was shown that the *Lactobacillales* bacterial order was a predominant faecal flora in cows on a SARA‐inducing diet (Mao et al., 2012). The SGD including *Enterococcus* spp. and *Streptococcus bovis* are mainly lactic acid producers and common intestinal and faecal flora in ruminants that can also involve in the LAB category (Devriese et al., 1992; Ghali et al., 2004). Despite the falling trend in SGD organisms in the rumen, its faecal count showed a non‐significant rising trend during SARA in the studied lambs. Incompatible with our results, performing a SARA‐inducing challenge reduced the number of *Streptococcus bovis* in caecal digesta and faeces of dairy cows (Plaizier et al. (2017b). The differences in species (sheep versus cow), diagnostic techniques (culture‐based versus molecular methods), consumed concentrate (crushed corn versus wheat and barley), and SARA duration might explain this discrepancy. Moreover, it has been reported that compared with rumen pH perturbation, the bacterial population changes during SARA in sheep were more stable and valuable for SARA screening (F. Li et al., 2017). Therefore, understanding the changes in rumen and faecal bacterial populations in lambs on a SARA‐inducing diet might be conducive to identifying the risk prediction or diagnosis of SARA, establishing appropriate nutritional strategies, and applying in situ treatment and control methods in sheep flock.

## 5 CONCLUSIONS

As an interesting finding, SARA in the lambs did not increase serum LPS value; it may be attributed to the short duration of SARA in the studied lambs or probable high clearance of LPS by the liver in the lambs. However, this necessitates more research for a more definitive conclusion.

The increased rumen (4.10 ± 2.80 × 10^7^ CFU/ml) and faecal (3.60 ± 2.50 × 10^7^ CFU/g) LAB can be useful indicators for improving the nutritional strategy, developing flock health practice, and screening SARA in lambs. In addition, SARA had no significant effects on blood LPS and lactate levels in the lambs.

Given the strong positive correlation observed between the rumen pH and the faecal pH changes, a reduced faecal pH at 6.30 ± 0.20 level could also be helpful for predicting SARA risk in lambs.

## AUTHOR CONTRIBUTIONS

*Conceptualization, methodology, formal analysis, writing–original draft, and writing–review and editing*: Ali Abbas Nikvand. *Conceptualization, methodology, and writing–review and editing*: Mohammad Nouri. *Investigation and data curation*: Rahman Rakhshandeh. *Investigation, methodology, and data curation*: Darioush Gharibi.

## CONFLICTS OF INTEREST

The authors declare no conflict of interest.

### ETHICS STATEMENT

This research was approved by the Ethics Committee in the Faculty of Veterinary Medicine, Shahid Chamran University of Ahvaz, Iran (approval No. EE/97.24.3. 70204/scu.ac.ir).

### PEER REVIEW

The peer review history for this article is available at https://publons.com/publon/10.1002/vms3.978

## Data Availability

Data are available upon reasonable request to the corresponding author.
